# Graphene Oxide-Polymer Composite Langmuir Films Constructed by Interfacial Thiol-Ene Photopolymerization

**DOI:** 10.1186/s11671-017-1864-8

**Published:** 2017-02-08

**Authors:** Xiaona Luo, Kai Ma, Tifeng Jiao, Ruirui Xing, Lexin Zhang, Jingxin Zhou, Bingbing Li

**Affiliations:** 1 State Key Laboratory of Metastable Materials Science and Technology, Yanshan University, Qinhuangdao, 066004 People’s Republic of China; 2 Hebei Key Laboratory of Applied Chemistry, School of Environmental and Chemical Engineering, Yanshan University, Qinhuangdao, 066004 People’s Republic of China; 3 Institute of Process Engineering, State Key Laboratory of Biochemical Engineering, Chinese Academy of Sciences, Beijing, 100190 People’s Republic of China; 4 Department of Chemistry and Biochemistry, Central Michigan University, Mount Pleasant, MI 48859 USA

**Keywords:** Graphene oxide, Thiol-ene photopolymerization, Langmuir film, Self-assembly, Composite film

## Abstract

The effective synthesis and self-assembly of graphene oxide (GO) nanocomposites are of key importance for a broad range of nanomaterial applications. In this work, a one-step chemical strategy is presented to synthesize stable GO-polymer Langmuir composite films by interfacial thiol-ene photopolymerization at room temperature, without use of any crosslinking agents and stabilizing agents. It is discovered that photopolymerization reaction between thiol groups modified GO sheets and ene in polymer molecules is critically responsible for the formation of the composite Langmuir films. The film formed by Langmuir assembly of such GO-polymer composite films shows potential to improve the mechanical and chemical properties and promotes the design of various GO-based nanocomposites. Thus, the GO-polymer composite Langmuir films synthesized by interfacial thiol-ene photopolymerization with such a straightforward and clean manner, provide new alternatives for developing chemically modified GO-based hybrid self-assembled films and nanomaterials towards a range of soft matter and graphene applications.

**Electronic supplementary material:**

The online version of this article (doi:10.1186/s11671-017-1864-8) contains supplementary material, which is available to authorized users.

## Background

Graphene oxide (GO)-based composites have received great attention over the past decade due to abundant oxygen-containing functional groups, which demonstrate GO moderate water dispersibility and reactive sites for further modification [1–4]. For examples, in recent years, Kim’s group has achieved excellent research works on the investigation of chemical modification of carbon-based materials and graphene nanocomposites as well as the applications for supercapacitors and liquid crystals [5–10]. In recent years, a lot of efforts have been devoted to rational design and controlled synthesis of various GO-based organic/inorganic nanocomposites, which promises better processability with electronic, optical, and electrochemical properties as well as great developments for nanomaterial applications [11–13]. The chemical modification and functionalization of GO materials with organic compounds are needed in order to make them appropriate for good dispersion and various applications [14–16]. For example, various small molecules including long-chain alkylamine, isocyanate derivatives, porphyrin, dopamine, or tetrathiafulvalene can be utilized to modify GO to obtain good dispersed in solvent and useful optical properties [17–21]. In comparison with small molecules, polymers can be also employed to modify GO to improve the properties of GO-based composites in distinct domains. For example, poly(2-(dimethylamino) ethyl methacrylate) and poly(vinyl alcohol) have been utilized to functionalized GO with different chemical reactions [22, 23]. However, this strategy more or less suffers from GO sheet aggregation, incomplete adsorption of organic molecules, and undesired side effects, which is unfavorable for subsequent desired applications. Alternatively, click chemistry has attracted great development in recent years due to its modular nature, high selectivity, and yields [24–27]. In contrast to click chemistry for Cu(I) system [28], no catalyst is required as the reaction is initiated thermally or photochemically. It is reported that the click chemistry has been displayed to functionalize GO with the thiol-ene/thiol-yne reactions or the azide-alkyne reactions [29–32]. Hence, it remains a formidable challenge to directly synthesize GO-based composites with eco-friendly and condition-gentle process in an effective organized self-assembly manner. On the other hand, Langmuir and Langmuir-Blodgett (LB) techniques are well known as a sophisticated and effective way in organizing molecules or building blocks in a two-dimensional confined environment to obtain interfacial organized films [33–35].

Since the initial reports about GO self-assembly in Langmuir films from Huang’s group [36, 37], some recent studies present the successful preparation of GO monolayer or GO-based composite films using the LB assembly method [38–46]. It can be expected that a combination of GO-based composites involved in click chemistry and self-assembly films by Langmuir technique should be particularly advantageous owing to their excellent biocompatibility, moderate nanostructures, and enhanced mechanical and chemical properties. To the best of our knowledge, GO-polymer composite films, particularly Langmuir films by interfacial thiol-ene photopolymerization, have not yet been reported and obtained. Thus, aqueous soluble poly(ethylene glycol) diacrylate (abbreviated as DA) containing a number of ethylene glycol as molecular skeleton and ene residues as headgroups capable of donating photopolymerization has been chosen for the formation of composite films.

**Fig. 1:**
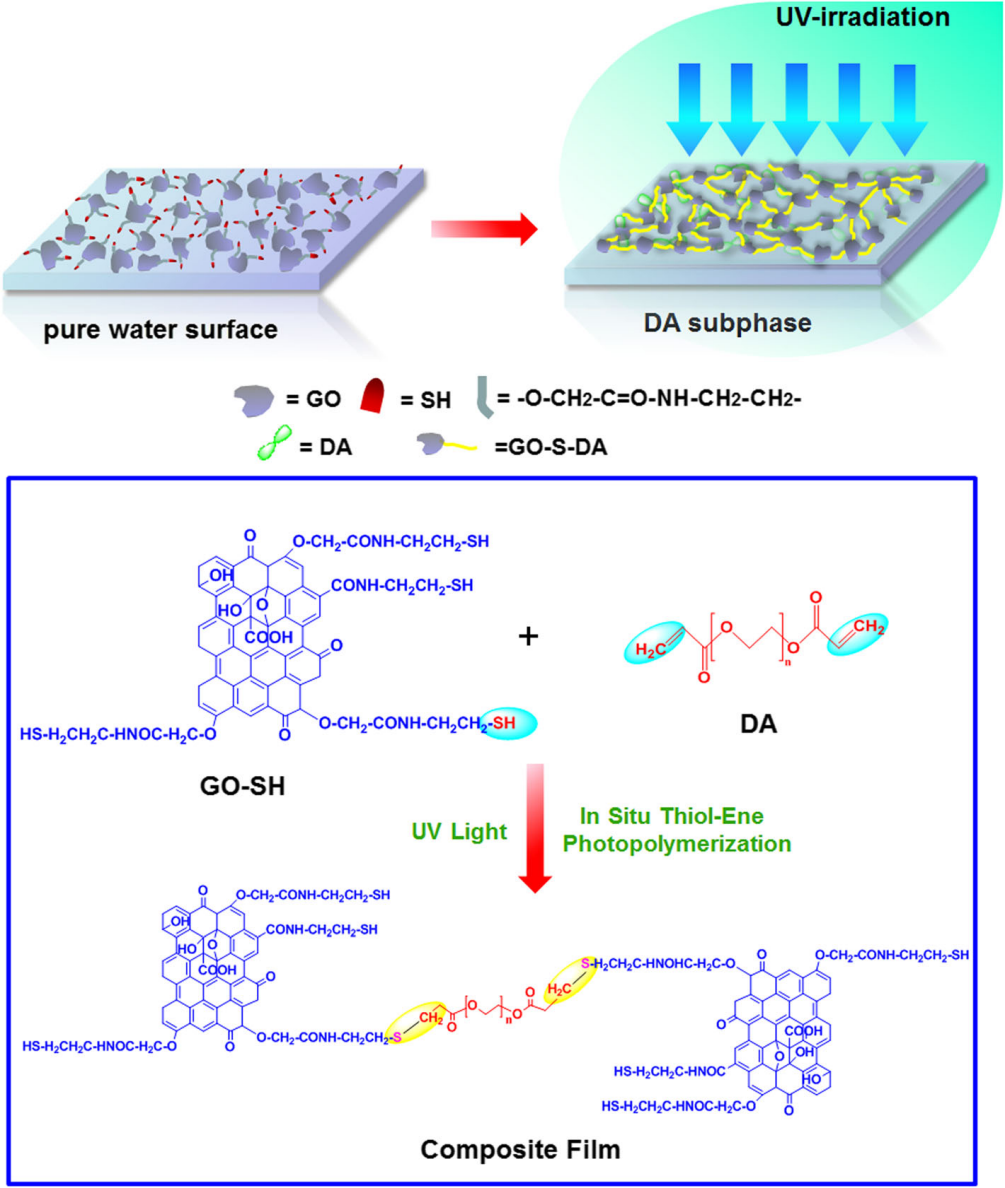
Scheme illustration of GO-SH composite Langmuir films constructed by interfacial thiol-ene photopolymerization

Herein, we report the synthesis of stable GO-polymer composite Langmuir films by in situ interfacial thiol-ene photopolymerization at room temperature (Fig. 1), without the use of any crosslinking and stabilizing agents by a facile and effective manner. We discover that photopolymerization reaction between thiol groups modified GO sheets and ene in polymer molecules is responsible for formation of such kind of composite films. Also, the as-prepared films can be easily transferred onto different supported substrates with assistance of the Langmuir-Blodgett (LB) assembly technique, which show potential to enhance mechanical and chemical properties and promote excellent biocompatibility. Thus, the present GO-polymer composite Langmuir films will provide significant potential for application in soft matter engineering and GO functionalization.

## Methods

The experimental used materials, poly(ethylene glycol) diacrylate (*M*_*w*_ = 1000 g mol^−1^, abbreviated as DA), cysteamine (95%, abbreviated as CA), and chloroacetic acid were purchased from Aladdin Reagent (Shanghai, China). Graphite powder (325 mesh, 99%) was purchased from Alfa Aesar Chemicals (Shanghai, China). N-(3-dimethylaminopropyl)-N′-ethylcarbodiimide hydrochloride (EDC-HCl) and N-hydroxysuccinimide (NHS) were purchased from Sigma-Aldrich and were used without purification. Sulfuric acid (H_2_SO_4_, 98%), potassium permanganate (KMnO_4_), potassium nitrate (KNO_3_), hydrogen peroxide (H_2_O_2_, 30%, *w*/*w*), and hydrochloric acid (HCl) were purchased from Beijing Chemicals or Tianjin KaiTong Chemicals and used without further purification. All aqueous solutions were prepared with water purified in a double-stage Milipore Milli-Q Plus purification system. Firstly, graphene oxide (GO) was prepared from graphite powder by a modified Hummers method [47]. Then, the carboxyl-functionalized GO (named as GO–COOH) was prepared according to the reported literature [48] and freeze dried in low temperature −50 °C). Thiol functionalization was done following analogous procedure described in the literatures [49, 50] and modified as described in the following. The obtained dispersion of exfoliated GO–COOH (100.0 mL of 1.5 mg/mL) was mixed with 2.55 g of NHS and 4.28 g of EDC-HCl into a round flask, under ice bath. After stirring vigorously for 2 h, 3.0 g of CA was added to the mixture and left stirring overnight under ice bath and next 3 days at room temperature. The resulting thiol-modified GO solid (named as GO-SH) was separated by filtration by using an amide membrane filter (0.2 μm), repeatedly washed with deionized Milli-Q water. Then, the solid was dispersed with water and dialyzed in water for 4–5 days with dialysis tubing (MWCO 12400). After dialysis, GO-SH powder was obtained by freeze-drying.

The interfacial characterization and LB film transfer were carried out in KSV-NIMA Minitrough LB system. The trough was carefully cleaned with chloroform and ethanol and then filled with DI water or aqueous DA solution. After a sonication of 10 min, some volumes of GO-SH spreading solution (0.504 mg/mL, different mixed solvent ratios and volumes) were dropwise spread onto pure water surface or DA subphase solution (0.500 g/L) using a glass syringe. Surface pressure was monitored using a tensiometer attached to a Wilhelmy plate. The film was compressed by barriers at a speed of 2 mm/min. For the Langmuir film of GO-SH on DA subphase by photoreaction, UV light of 365 nm was irradiated on interfacial films at 5 min before compression begin and kept through whole compress process. The GO-SH monolayer was transferred to substrates at various points during the compression by vertically dipping the substrate into the trough and slowly pulling it up (2 mm/min). Mica, quartz, and, CaF_2_, glass plates were used as the substrates to transfer monolayer or multilayer for the next morphological and spectral characterizations. Quartz and glass plates were treated with 1:1:5 NH_4_OH:H_2_O_2_:H_2_O (by volume) and washed repeatedly with deionized water before use.

The morphology of composite films were characterized by using a field-emission scanning electron microscopy (FE-SEM, S-4800II, Hitachi, Japan) with the accelerating voltage of 5–15 kV. The chemical composition of the samples was characterized by energy-dispersive X-ray spectroscopy (EDXS). EDXS analysis was typically performed at an accelerating voltage of 200 kV, using an Oxford Link-ISIS X-ray EDXS microanalysis system attached to SEM. Atomic force microscopy (AFM) images were measured by using a Nanoscope model MultiMode 8 Scanning Probe Microscope (Veeco Instrument, USA) with silicon cantilever probes. Raman spectroscopy was performed using a HORIBA Jobin Yvon XploRA PLUS confocal Raman microscope equipped with a motorized sample stage. The wavelength of the excitation laser was 532 nm, and the power of the laser was kept below 1 mW without noticeable sample heating. The intensity of a Raman peak was extracted from the maximum value after baseline subtraction over corresponding spectral range. X-ray photoelectron spectroscopy (XPS) was performed on the Thermo Scientific ESCALab 250Xi using 200 W monochromated Al Kα radiation. The 500 μm X-ray spot was used for XPS analysis. The base pressure in the analysis chamber was about 3 × 10^−10^ mbar. Typically, the hydrocarbon C1s line at 284.8 eV from adventitious carbon is used for energy referencing. Both survey scans and individual high-resolution scans for S(2p), O(1s), and C(1s) peaks were recorded. FTIR spectra were recorded on a Fourier infrared spectroscopy (Thermo Nicolet Corporation) by the conventional KBr disk tablet method or composite films on CaF_2_ plates. X-ray diffraction study was carried out by using an X-ray diffractometer (SmartLab, Rigaku) equipped with a conventional Cu Kα X-ray radiation (*λ* = 1.54 Å) source and a Bragg diffraction setup. Elemental analysis was carried out with a FlashEA Carlo-Erba-1106 Thermo-Quest. A UV lamp (20 mW/cm^2^, *λ* = 365 nm; LUYOR-3405; LUYOR Corporation) was used to irradiate the Langmuir films to perform the photochemical reactions.

## Results and Discussion

The thiol-functionalized GO-SH material can serve as building blocks for preparation of Langmuir films and subsequent in situ interfacial thiol-ene photopolymerization under UV light irradiation by standard Langmuir self-assembly technique. Figure 1 demonstrates the illustration of GO-SH composite Langmuir films constructed by in situ thiol-ene photopolymerization. For this, the precursor GO, the intermediate GO–COOH, and the final building block GO-SH were synthesized and characterized by many methods. XRD curves of as-prepared materials show the disappearance of Bragg peaks at 2*θ* value of 11.2° assigned to the (001) diffraction peak and presence of new broad peak at 2*θ* value of 22.1° for GO-SH (Additional file 1: Figure S1), which indicate successful thiolation in GO sheet and 3D structural composite formation due to thiol-functionalization [49]. The elemental analysis data (Additional file 1: Table S1) of as-prepared GO-based materials show the obvious increment of carbon composition and appearance of sulfur component with value of 10.10 ± 0.07 wt.% for obtained GO-SH. The comparison of XPS data and elemental analysis data show similar calculated N/C ratios and S/C ratios (Additional file 1: Table S2), which is almost in agreement with the above obtained results. Thus, we inspected the interfacial phase behaviors and characterized the Langmuir and the transferred LB films of GO-SH by means of morphological and spectral methods.

**Fig. 2:**
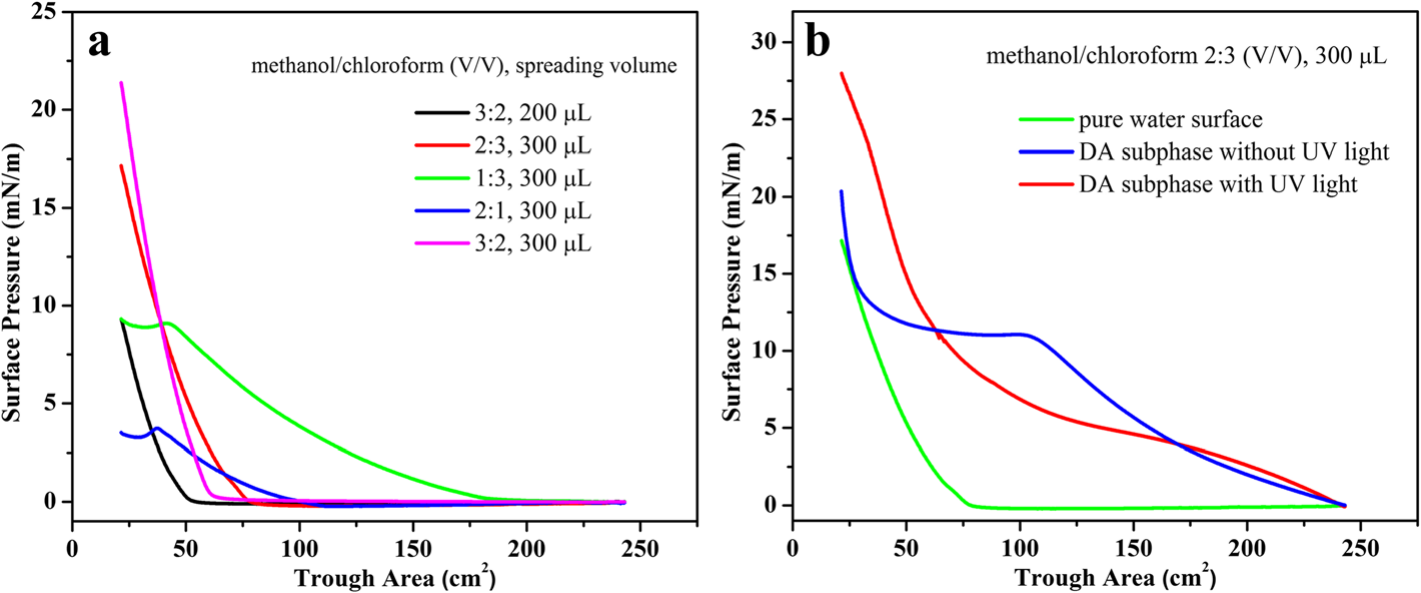
Surface pressure-area isotherms of Langmuir films of as-prepared GO-SH solution: **a** pure water surface, change of different mixed solvent ratios and volumes; **b** DA subphase with/without UV light at preferred spreading condition

Firstly, we measured the surface pressure-area isotherms of as-prepared Langmuir films at room temperature (Fig. 2). The optimization of used different spreading mixed solvents and volumes for GO-SH Langmuir film spread on pure water surface was demonstrated in Fig. 2a. It show that the optimized spread condition (methanol/chloroform 2:3 (*V*/*V*), 300 μL) seem suitable for the next interfacial characterization. Then, as shown in Fig. 2b, it can be easily observed that the isotherm of GO-SH spread on pure water surface show slow increment of pressure at trough area of 77 cm^2^ until reaching 18 mN/m at compression end of trough area 23 cm^2^. For the GO-SH film on DA subphase without UV light irradiation, the obtained curve demonstrate increment of pressure at the beginning of compression and long platform region after 11 mN/m, suggesting possible conformation change of building block GO-SH due to the adsorption process with DA molecules in subphase solution. In addition, for the GO-SH film on DA subphase with UV light irradiation, the platform region disappears and the final surface pressure increase to the value of 28 mN/m. The obvious change in isotherms suggests the occurrence of interfacial thiol-ene photopolymerization between thiol groups in GO-SH and DA molecules with UV light irradiation and formation of composite Langmuir films.

**Fig. 3:**
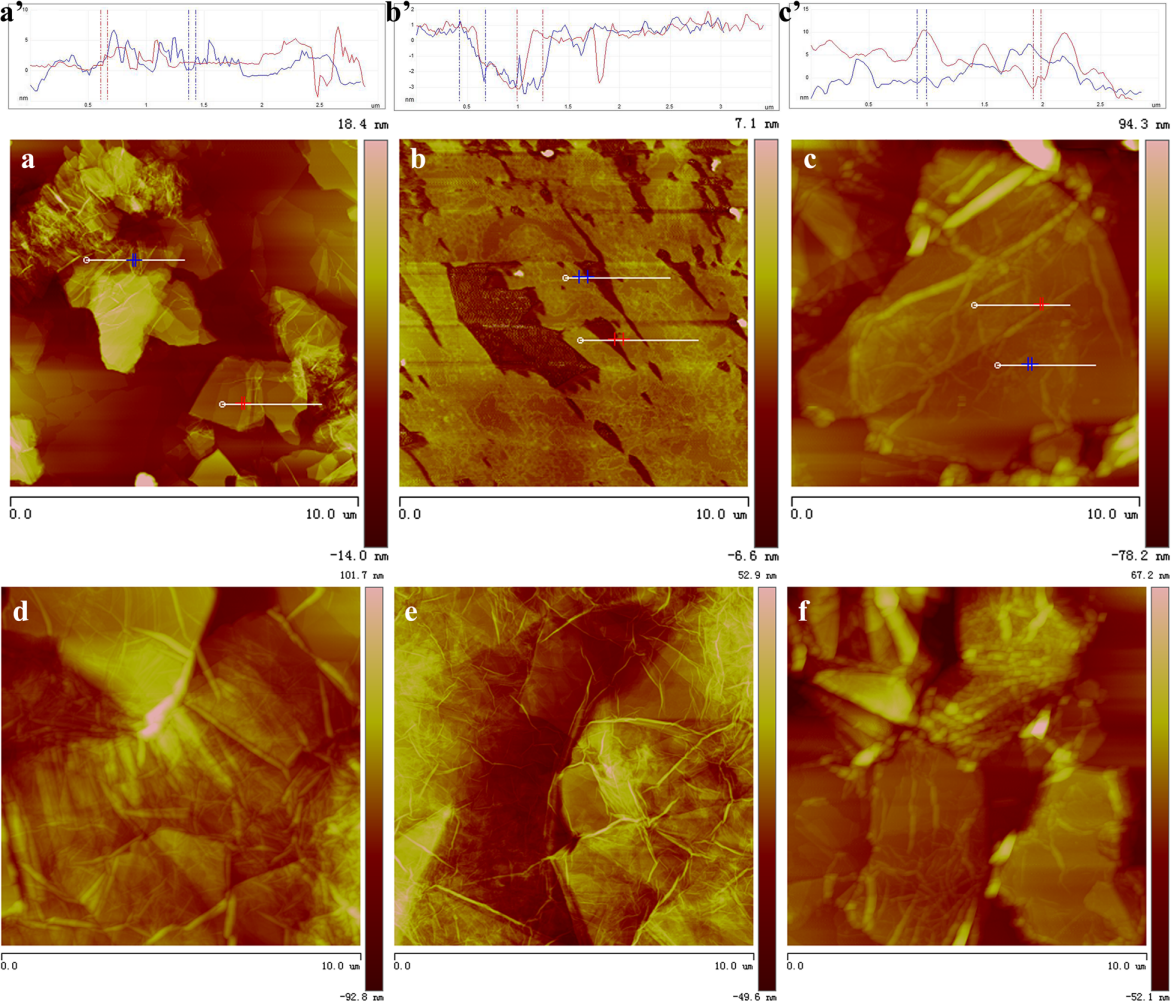
AFM images with section analysis (**a**’–**c**’) of monolayer Langmuir films of GO-SH spread on pure water surface (**a**, **d**), DA subphase without UV light (**b**, **e**), and with UV light by interfacial thiol-ene photopolymerization (**c**, **f**). The transferred surface pressures are 5 mN/m for images (**a**–**c**) and 15 mN/m for images (**d**–**f**)

**Fig. 4:**
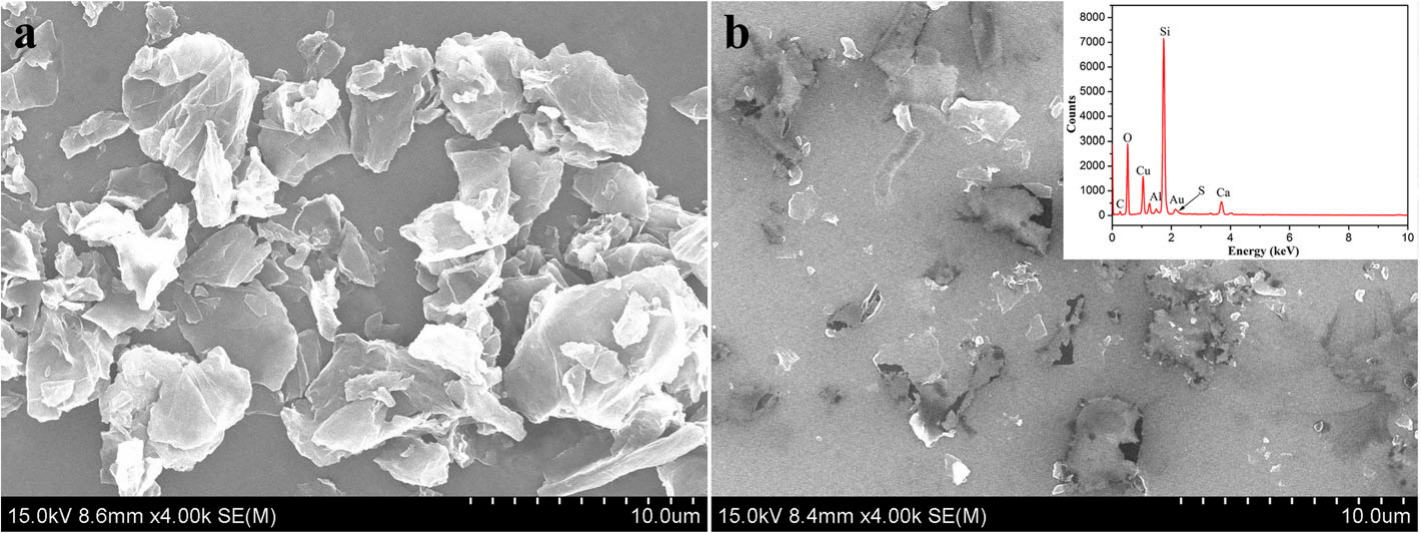
SEM images with EDX spectra analysis of transferred 10-layered multilayer LB films of GO-SH from pure water surface (**a**) and DA subphase with UV light by interfacial thiol-ene photopolymerization (**b**)

The morphologies of the nanostructures of monolayer GO-SH Langmuir films were characterized by AFM (Fig. 3). The formed different nanostructures are obviously observed. Firstly, at low surface pressure of 5 mN/m, the thiol-functionalized GO sheet fabricate the GO-SH Langmuir films with the height distribution of 1.2 ± 0.2 nm (Fig. 3a’). When from DA subphase without UV light, films with small aggregates on surface dominate with the main height distribution of 3.4 ± 0.3 nm (Fig. 3b’), suggesting the possible interfacial adsorption of DA molecules on GO sheet due to hydrophilic force and Van der Waals force or bilayer film formation due to conformation change of GO-SH. In addition, as for the film from DA subphase with UV light, the nanostructures become cluttered in three dimensional modes and some strip-like aggregates appear on GO sheet (Fig. 3c), suggesting the interfacial thiol-ene photopolymerization between thiol groups in GO-SH and DA molecules. In addition, with the increment of surface pressure of 15 mN/m, closely packed self-assembled films with obviously increased thickness can be formed, as shown in Fig. 3d–f. The difference of morphologies and height between the GO-SH Langmuir films from various subphases can be mainly due to the introduction of the DA molecules in the composite Langmuir film. In addition, we also compared the morphologies of multilayer LB films of GO-SH from pure water and DA subphase with UV light by thiol-ene photopolymerization. It is clearly observed from SEM images in Fig. 4 that the Langmuir films from pure water show dispersed GO-SH sheets, while the films from DA subphase by photopolymerization reaction demonstrate crosslinked films with embedded GO sheets.

**Fig. 5:**
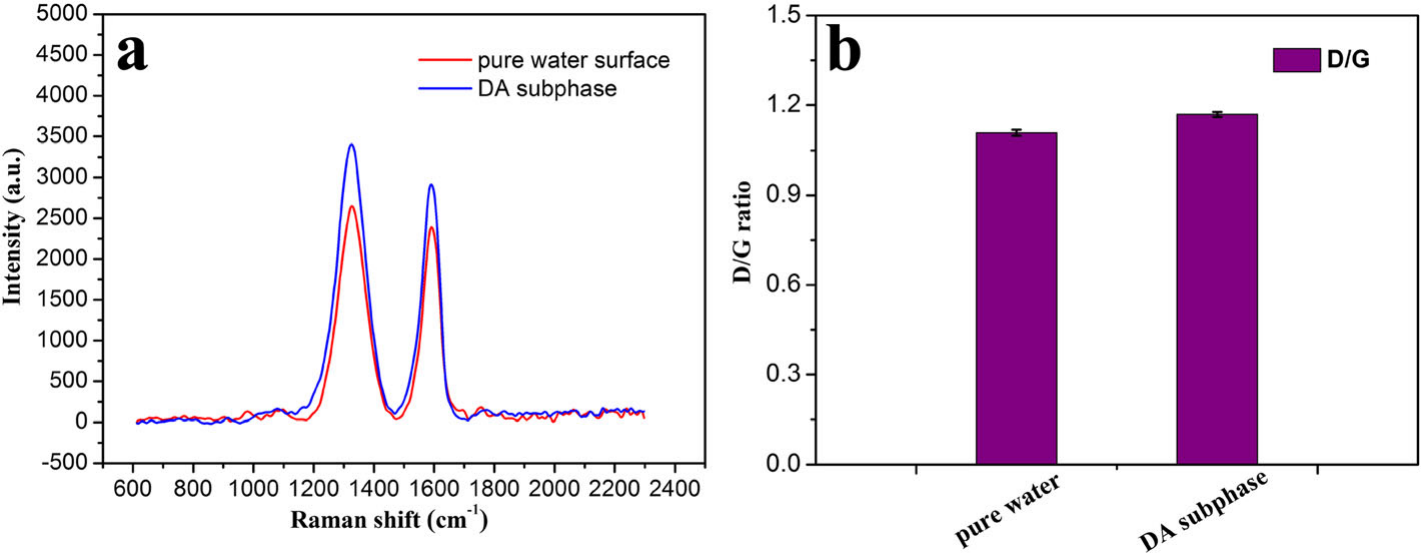
Raman spectra (**a**) and D/G ratio analysis (**b**) of transferred 40-layered LB films of GO-SH from pure water surface and DA subphase by interfacial thiol-ene photopolymerization

It is well known that Raman spectroscopy has been widely applied to characterize GO and relative composite materials. The measurement for the present obtained multilayer LB films of GO-SH from pure water surface and DA subphase by in situ thiol-ene photopolymerization was performed. Two characteristic bands of graphene sheets in Raman spectra appear in Fig. 5a. One band at 1601 cm^−1^ can be assigned to the G band, which originates from the first-order scattering of the E_2_g phonons of the sp^2^-hybridized carbon atoms. Another band at 1351 cm^−1^ can be attributed to the D band, which comes from a breathing mode of κ-point phonons of A_1_g symmetry of the defects involved in the sp^3^-hybridized carbon bonds such as hydroxyl and/or epoxide bonds [51, 52]. It was reported that the D/G peak intensity ratio could be used as a measurement of the sp^2^ domain size of graphene sheets containing sp^3^ and sp^2^ bonds due to the origination of G and D bands [53, 54]. For the present system in Fig. 5b, the D/G ratio shifted from 1.09 for LB films from water surface to 1.18 for LB films from DA subphase by in situ thiol-ene photopolymerization. The obvious change confirmed the successful photopolymerization reaction of polymeric DA molecules with thiol groups linked to GO sheet in the Langmuir films. In addition, the analysis of measured Raman spectra for the prepared GO-SH powder (Additional file 1: Figure S2) shows the calculated D/G ratio value of 1.05 in comparison with 0.91 and 0.92 for GO and GO–COOH materials.

**Fig. 6:**
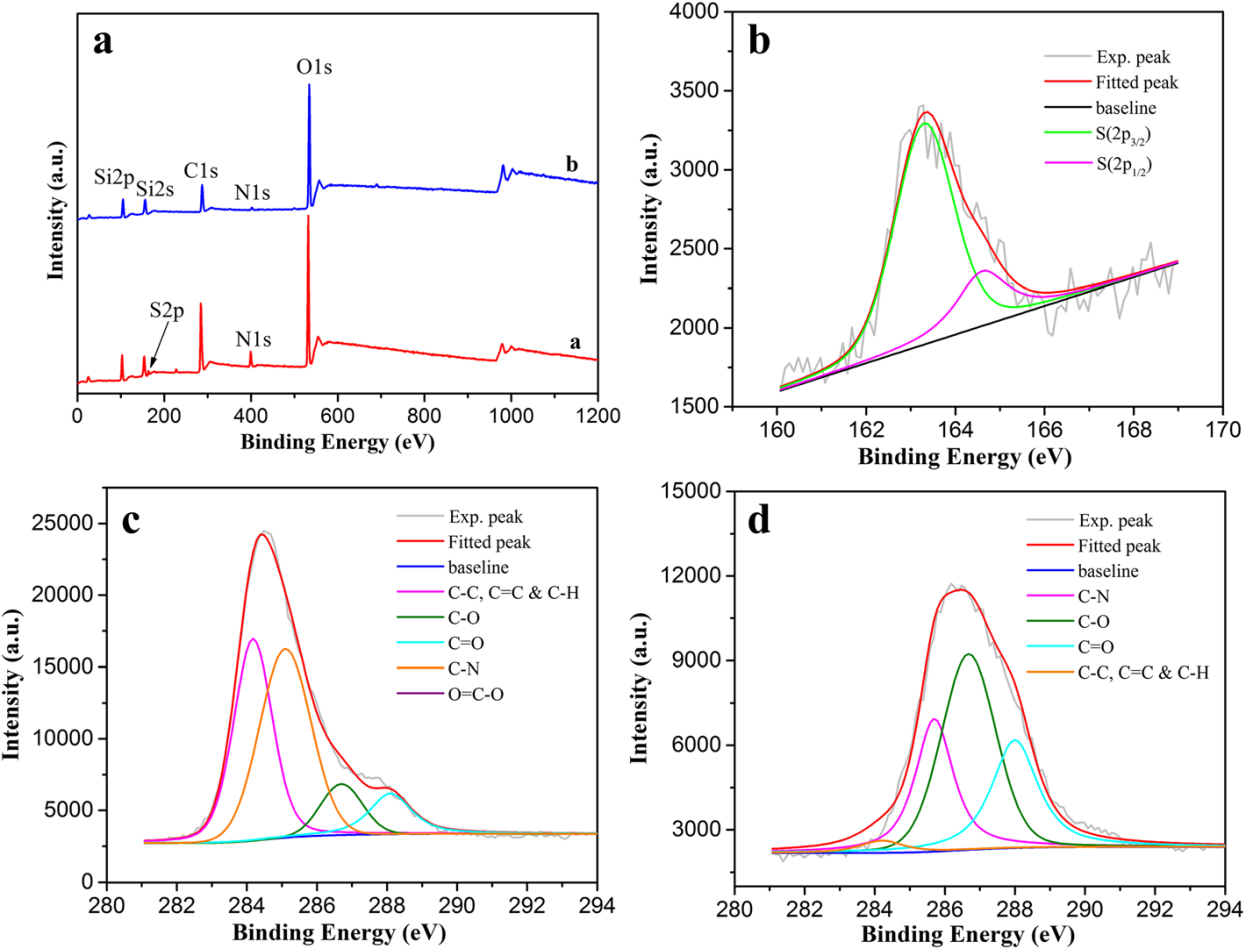
Survey XPS spectra (A) of transferred 40-layered LB films of GO-SH on pure water surface (**a**) and DA subphase by interfacial thiol-ene photopolymerization (**b**). Deconvolution of XPS peaks of films on pure water surface (B, S(2p); C, C(1s)), and DA subphase by interfacial thiol-ene photopolymerization (D, C(1s))

To characterize the composition and chemical state of different LB films, we performed X-ray photoelectron spectroscopy (XPS) measurements (Fig. 6). The survey data for GO-SH LB films demonstrate the characteristic C(1s), O(1s), and S(2p) peaks (Fig. 6a). In addition, obvious S(2p_3/2_) and S(2p_1/2_) peaks appear at the center positions of 163.3 and 164.6 eV, respectively (Fig. 6b), which can be assigned to the dominant C–SH and small amount of C–S–C bond [55–57]. Thus the majority of sulfur element in LB films (about 83%) is displayed to be covalently attached to GO in thiol group. The deconvolution of C(1s) peaks from the films on pure water surface in Fig. 6c demonstrates the peaks centered at positions of 284.2, 285.1, 286.7, and 288.1 eV, which can be assigned to the C–C, C=C, and C–H bonds, C–N, C–O, and C=O–O oxygen-containing bonds, respectively [58–60]. After in situ thiol-ene photopolymerization, the deconvolution C(1s) peaks attributed to C–O and C=O–O bonds showed obvious increment (Fig. 6d). Moreover, according to the analysis data in Table 1, the N/C ratio and S/C ratio are calculated to decrease 25 and 51% after thiol-ene photopolymerization, indicating the addition of carbon element composition and successful preparation of GO-polymer composite Langmuir films. In addition, the survey XPS spectra of all as-prepared lyophilized (Additional file 1: Figure S3A) demonstrate the additional characteristic N(1s) and S(2p) peaks in GO-SH powder compared with spectra of GO and GO–COOH. The deconvolution analysis of C(1s) peaks in GO-SH (Additional file 1: Figure S3D) shows also the new peak centered at position of 285.2 eV assigned to the C–N bonds compared with those in GO and GO–COOH (Additional file 1: Figure S3B, C), which indicate the successful thiolation in GO sheets with cysteamine molecules.

**Table 1:** Analysis of XPS data of as-prepared multilayer GO-SH LB films from different subphase^a^

GO-SH LB Films	C [at.%]	N [at.%]	S [at.%]	N/C ratio [%]	S/C ratio [%]
Pure water	44.03	5.61	1.57	12.7	3.57
DA subphase with UV light	31.47	3.01	0.54	9.56	1.72

^a^Values calculated from integrated area in XPS data in Fig. 6

In order to evaluate the successful functionalization in GO sheet and characterize the prepared composite films, FT-IR spectra were measured. The spectra of GO and GO–COOH in Additional file 1: Figure S4 show typical bands due to skeletal vibrations of graphene domains at 1632 cm^−1^ and characteristic OH and C=O stretching at 3432 and 1737 cm^−1^, respectively [61–64]. As for spectrum of GO-SH, some new characteristic bands appeared, such as amide C=O stretching at 1640 cm^−1^, N-H bending at 3312 cm^−1^ and N-H stretching at 1548 cm^−1^, which indicated the effective functionalization of thiol groups. In addition, obvious changes were observed for the IR spectra of transferred multilayer LB films of GO-SH before and after thiol-ene photopolymerization with DA subphase (Additional file 1: Figure S5). The spectrum of GO-SH composite films after thiol-ene photopolymerization show obvious intensity increment for bands at 2921 and 2850 cm^−1^ (C–H modes of methylene moieties), 1727 cm^−1^ (C=O stretching), and 1640 cm^−1^ (amide C=O stretching), indicating the addition of cysteamine molecules in films and effective thiol-functionalization. Moreover, the measured XRD curves of multilayer LB films of GO-SH before and after thiol-ene photopolymerization reaction show similar broad Bragg peaks centered at 2*θ* value of 22.7° (Additional file 1: Figure S6), which suggest the retainment of 3D structures in composite films. Taken together, the supramolecular graphene oxide-polymer composite Langmuir films have been constructed by in situ thiol-ene photopolymerization, providing a potential for further application in GO functionalization and soft matter engineering.

## Conclusions

In summary, we have presented a one-step chemical preparation of graphene oxide-polymer composite Langmuir films by in situ thiol-ene photopolymerization reaction in a facile and effective manner. Thiol-functionalized graphene oxide alone serves as either a crosslinking agent or an amphiphile for the formation of composite Langmuir films. The obtained GO-SH-DA composite films are demonstrated by the presence of a 3D nanostructure with embedded GO sheets in crosslinked films. The mechanism for the formation of the stable composite films involves a chemical thiol-ene photopolymerization reaction of thiol groups modified on GO sheet with ene components in soluble polymer chains. The resulting GO-polymer composite films can be easily transferred onto a supported substrate with assistance of the LB assembly method. Owing to the specific mechanical and chemical properties of graphene oxide and polymer composition, the present prepared composite films will have great potentials for application of soft matter engineering and graphene self-assembled nanomaterials.

## Additional File

Additional file 1: Supporting information. (DOCX 5395 kb)
