# Supplementary material for: Graphene Oxide-Polymer Composite Langmuir Films Constructed by Interfacial Thiol-Ene Photopolymerization
Source: Nanoscale Res Lett. 2017 Feb 8;12:99. doi: 10.1186/s11671-017-1864-8 (PMC5307420; doi:10.1186/s11671-017-1864-8)
Supplement: Additional file 1: — Supporting information. (DOCX 5395 kb) [file 11671_2017_1864_MOESM1_ESM.docx]

**Supporting Information**

**Graphene Oxide-Polymer Composite Langmuir Films Constructed by Interfacial Thiol-Ene Photopolymerization**

Xiaona Luo^1,2#^, Kai Ma^2,#^, Tifeng Jiao^1,2,3*^, Ruirui Xing^2,3^, Lexin Zhang^2^, Jingxin Zhou^2^, Bingbing Li^4,*^

^1^State Key Laboratory of Metastable Materials Science and Technology, Yanshan University, Qinhuangdao 066004, P. R. China

^2^Hebei Key Laboratory of Applied Chemistry, School of Environmental and Chemical Engineering, Yanshan University, Qinhuangdao 066004, P. R. China

^3^State Key Laboratory of Biochemical Engineering, Institute of Process Engineering, Chinese Academy of Sciences, Beijing 100190, P. R. China

^4^Department of Chemistry and Biochemistry, Central Michigan University, Mount Pleasant, MI 48859, USA

**Table S1.** Elemental analysis data of as-prepared GO-based materials.

| Sample | C [wt.%] | H [wt.%] | N [wt.%] | S [wt.%] |
| --- | --- | --- | --- | --- |
| GO | 44.30 ± 0.02 | 2.01 ± 0.08 | -- | -- |
| GO-COOH | 44.70 ± 0.06 | 2.10 ± 0.07 | -- | -- |
| GO-SH | 49.87 ± 0.07 | 4.33 ± 0.04 | 9.74 ± 0.03 | 10.10 ± 0.07 |

**Table S2.** Comparison of XPS data and elemental analysis data of as-prepared GO-based materials.

| Sample | C [at. %] | C [wt. %] | N [at. %] | N [wt. %] | S [at. %] | S [wt. %] | N/C ratio [at./at.]% | S/C ratio [at./at.]% |
| --- | --- | --- | --- | --- | --- | --- | --- | --- |
| GO-SH^a^ | 68.13 | -- | 9.66 | -- | 5.99 | -- | 14.2 | 8.79 |
| GO-SH^b^ | 37.39^b^ | 49.87 | 6.26^b^ | 9.74 | 2.84^b^ | 10.10 | 16.7 | 7.61 |

a, values calculated from integrated area in XPS data in Fig. S3; b, values calculated from elemental analysis data in Table S1.


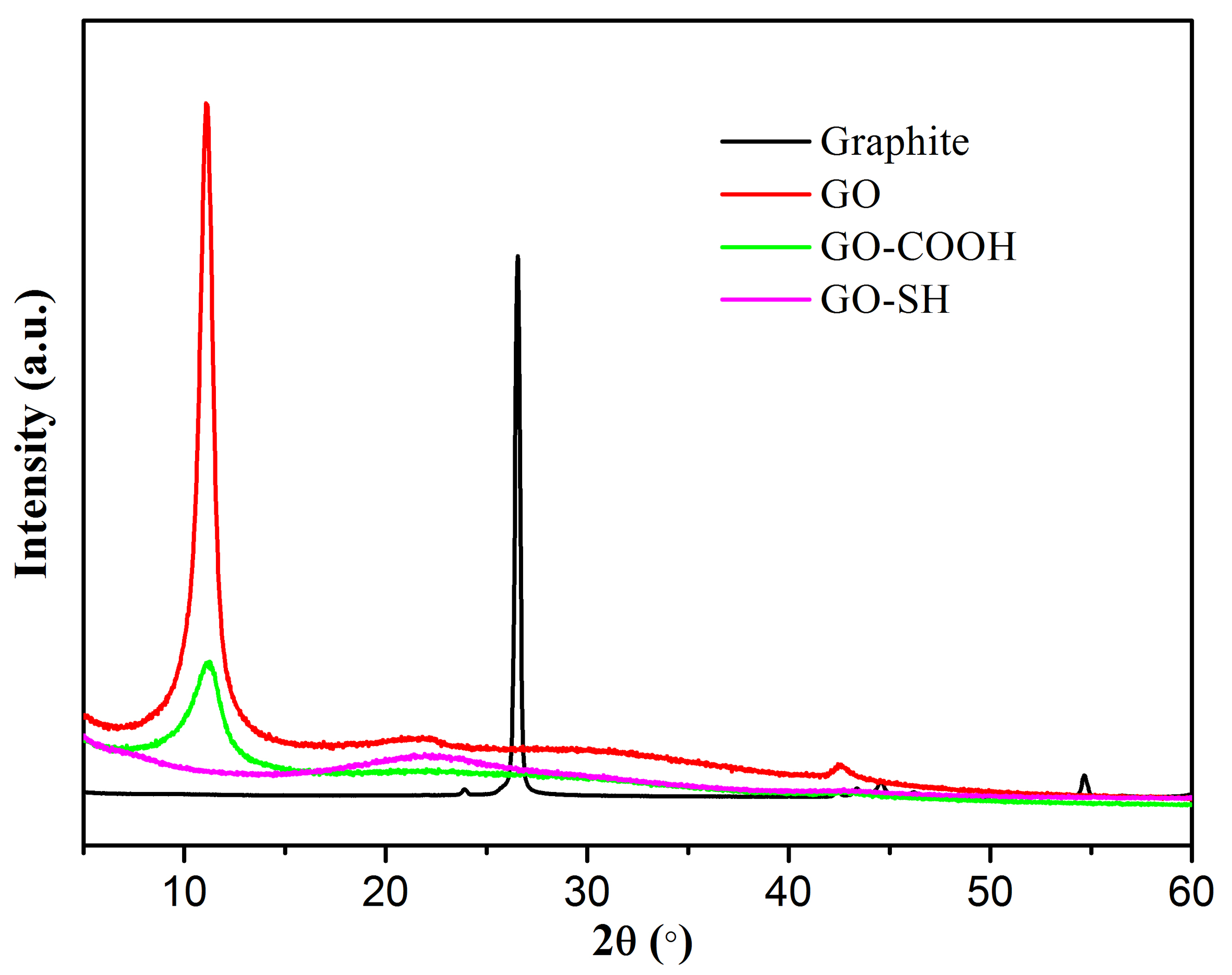


**Figure S1.** XRD curves of as-prepared materials: graphite, GO, GO-COOH, and GO-SH, respectively.


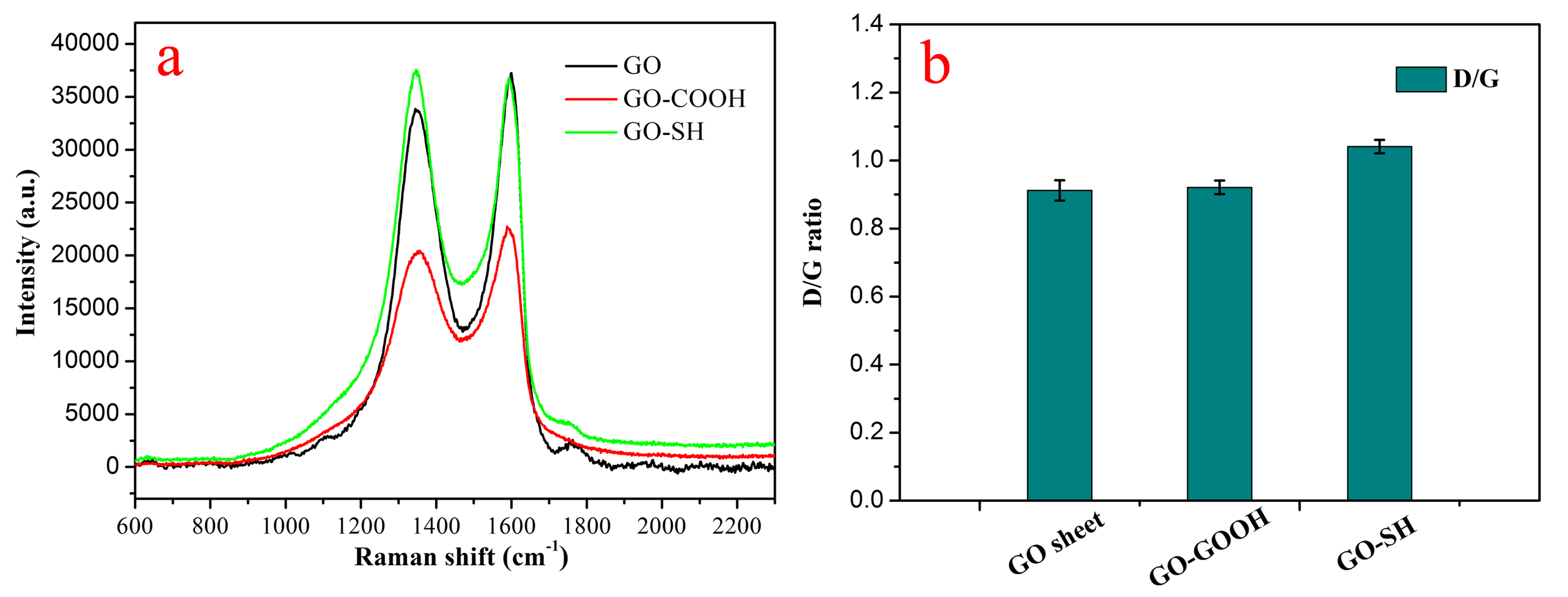


**Figure S2.** Raman spectra (a) and D/G ratio analysis (b) of as-prepared materials: GO, GO-COOH, and GO-SH, respectively.


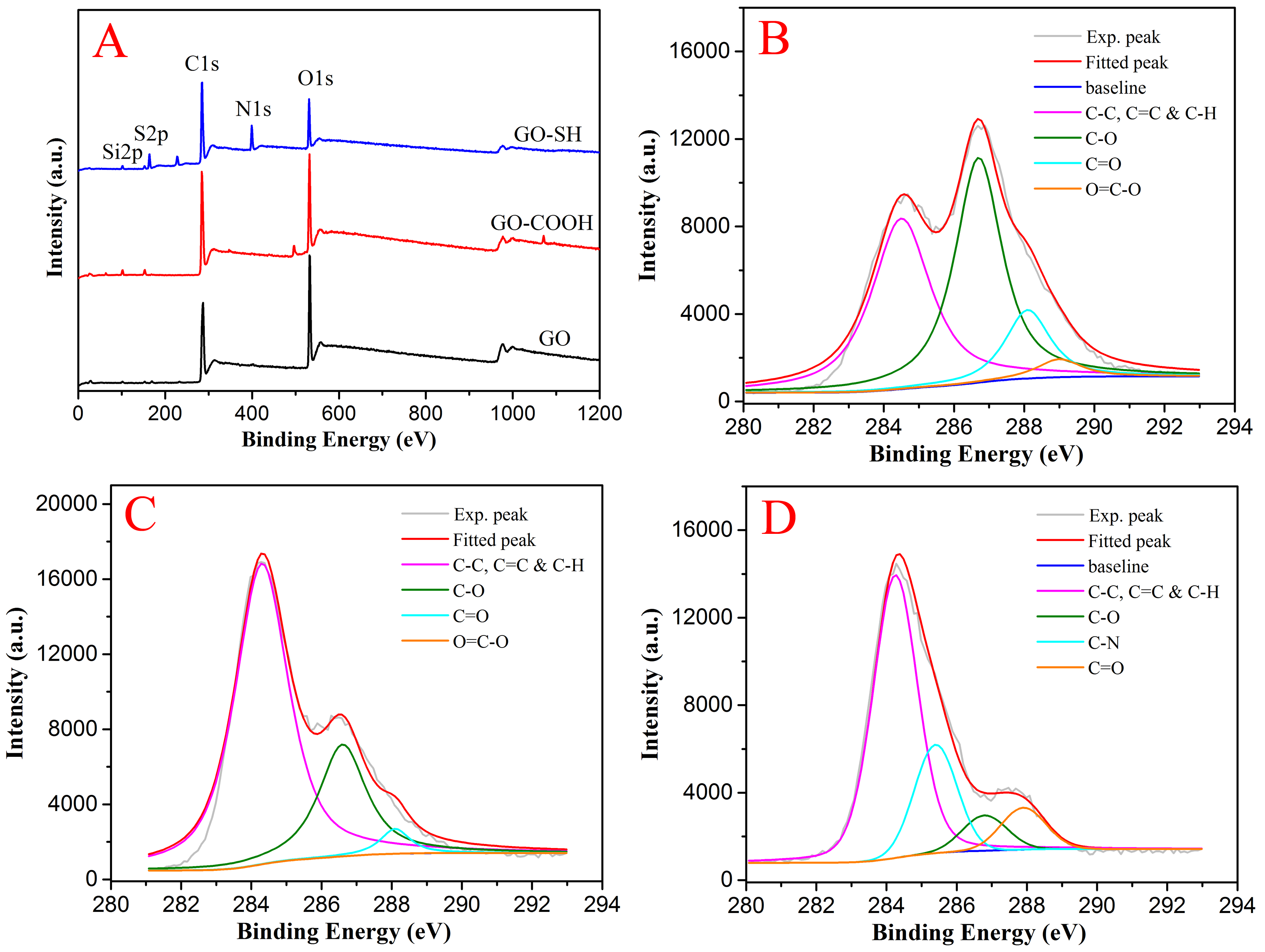


**Figure S3.** Survey XPS spectra (A) of present as-prepared lyophilized materials. Deconvolution of XPS C(1s) peaks of GO (B), GO-COOH (C), and GO-SH (D), respectively.


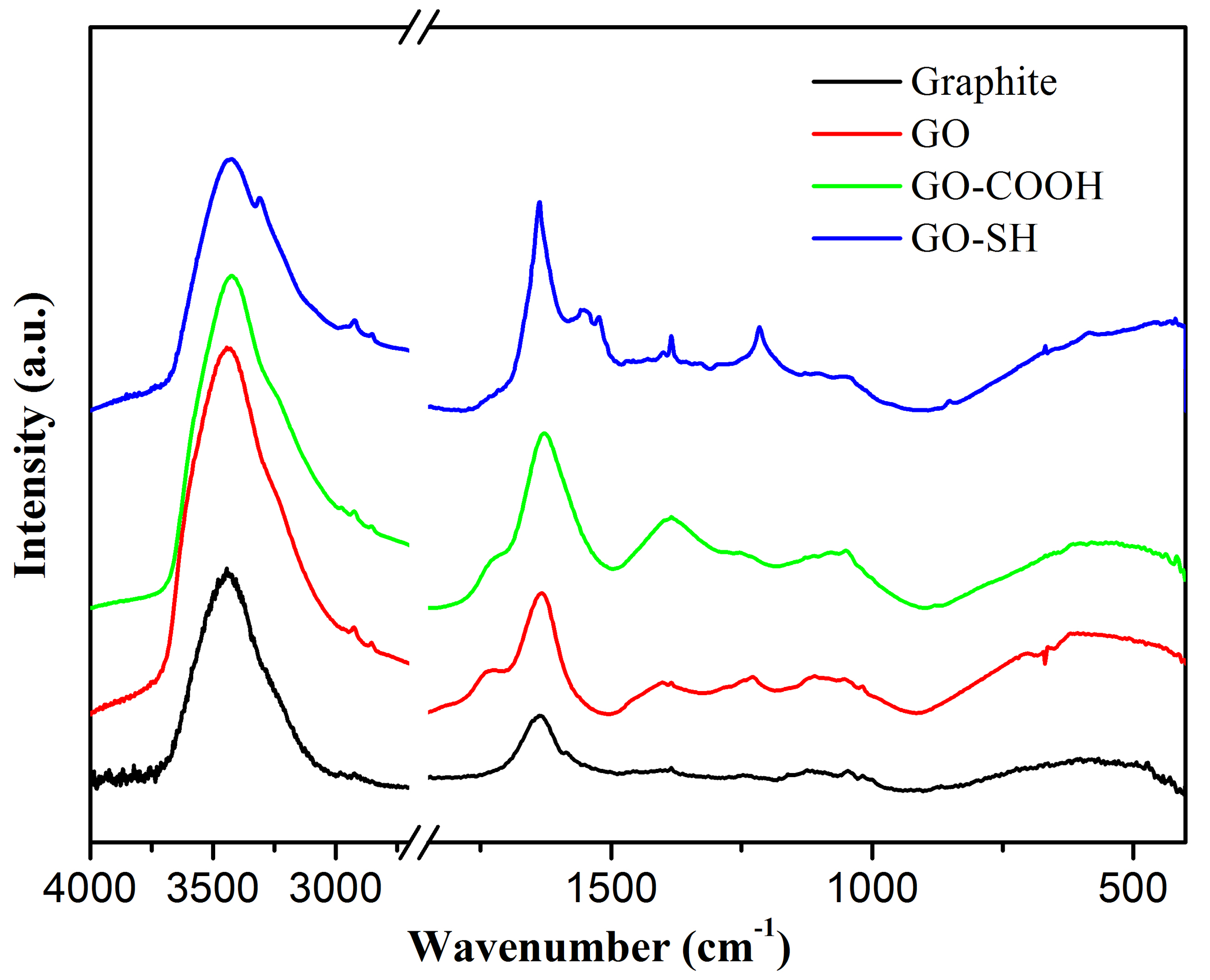


**Figure S4.** IR spectra of as-prepared materials: graphite, GO, GO-COOH, and GO-SH, respectively.


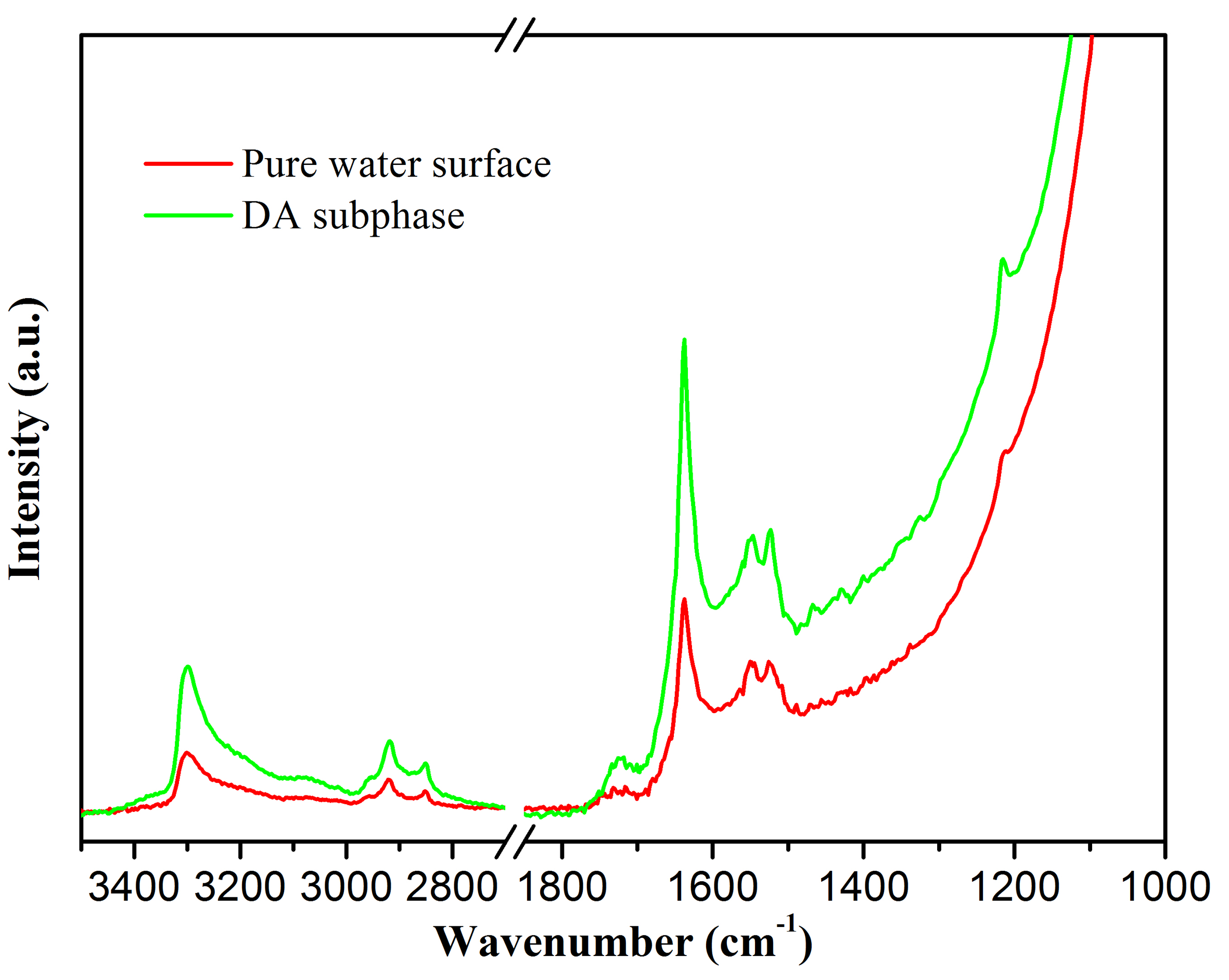


**Figure S5.** IR spectra of transferred 40-layered LB films of GO-SH from pure water surface and DA subphase by interfacial thiol-ene photopolymerization.


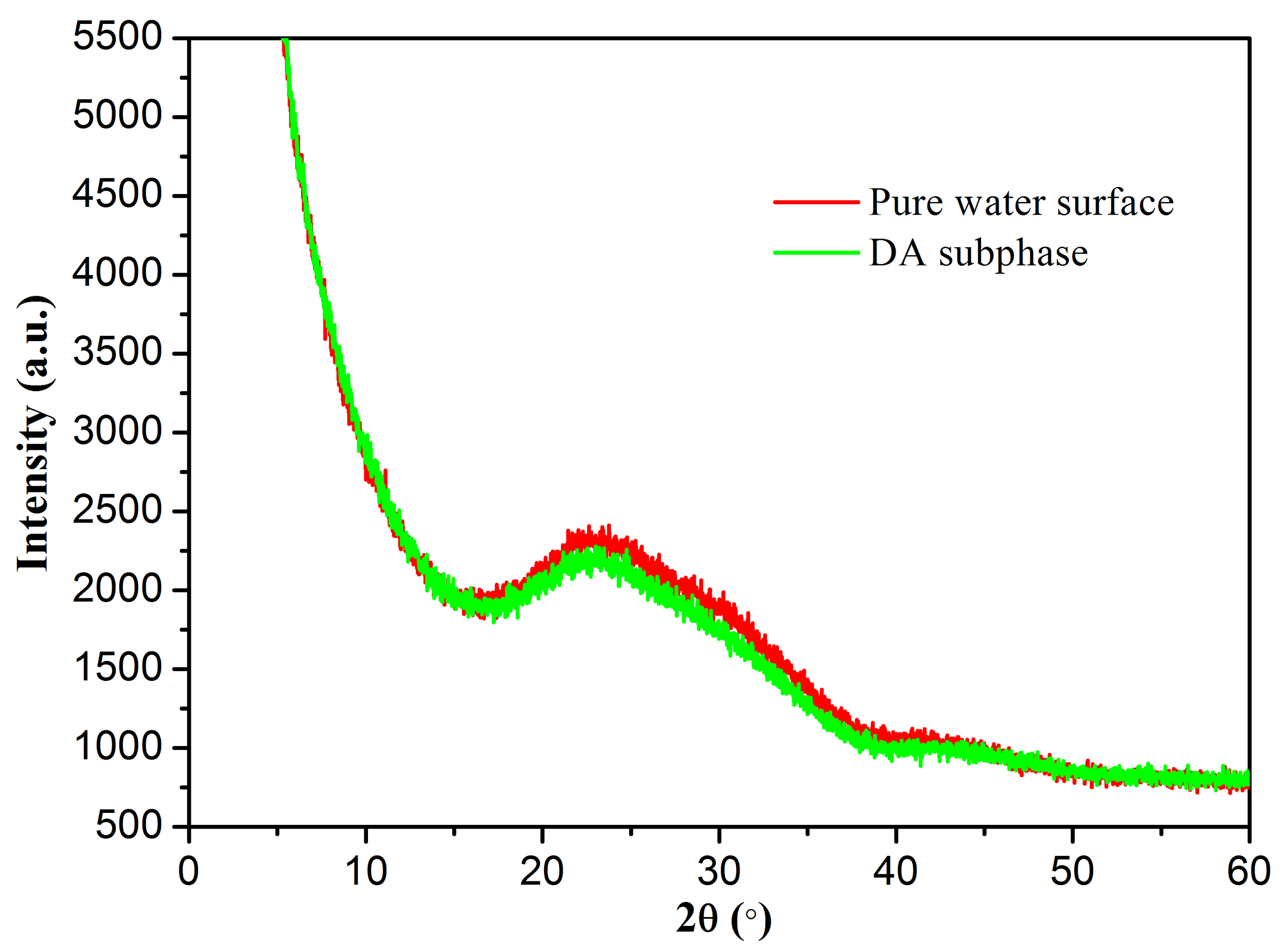


**Figure S6.** XRD curves of transferred 40-layered LB films of GO-SH from pure water surface and DA subphase by interfacial thiol-ene photopolymerization.
